# Cardioprotective potential of *N*-acetyl cysteine against hyperglycaemia-induced oxidative damage: a protocol for a systematic review

**DOI:** 10.1186/s13643-017-0493-8

**Published:** 2017-05-12

**Authors:** Phiwayinkosi V. Dludla, Bongani B. Nkambule, Stephanie C. Dias, Rabia Johnson

**Affiliations:** 1 Biomedical Research and Innovation Platform (BRIP), Medical Research Council, Francie van Zijl Drive, P.O. Box 19070, Tygerberg, 7505 South Africa; 2 School of Laboratory Medicine and Medical Sciences (SLMMS), College of Health Sciences, University of KwaZulu-Natal, Durban, South Africa; 3 Division of Medical Physiology, Faculty of Health Sciences, Stellenbosch University, Tygerberg, South Africa

**Keywords:** Diabetes mellitus, Cardiomyopathy, Oxidative stress, *N*-acetyl cysteine, *N*-acetylcysteine, Cardiac protection

## Abstract

**Background:**

Hyperglycaemia-induced oxidative damage is a well-established factor implicated in the development of diabetic cardiomyopathy (DCM) in diabetic individuals. Some of the well-known characteristics of DCM include increased myocardial left ventricular wall thickness and remodelling that result in reduced cardiac efficiency. To prevent this, an increasing number of pharmacological compounds such as *N*-acetyl cysteine (NAC) are explored for their antioxidant properties. A few studies have shown that NAC can ameliorate hyperglycaemia-induced oxidative damage within the heart. Hence, the objective of this review is to synthesise the available evidence pertaining to the cardioprotective role of NAC against hyperglycaemia-induced oxidative damage and thus prevent DCM.

**Methods:**

This systematic review protocol will be reported in accordance with the Preferred Reporting Items for Systematic Review and Meta-Analysis Protocols (PRISMA-P) 2015 statement. We will perform a comprehensive search on major databases such as EMBASE, Cochrane Library, PubMed and Google scholar for original research articles published from January 1960 to March 2017. We will only report on literature that is available in English. Two authors will independently screen for eligible studies using pre-defined criteria, and data extraction will be done in duplicate. All discrepancies will be resolved by consensus or consultation of a third reviewer. The quality of studies will be checked using Cochrane Risk of Bias Assessment Tool and The Joanna Briggs Institute (JBI) Critical Appraisal tools for non-randomised experimental studies. Heterogeneity across studies will be assessed using the Cochrane Q statistic and the inconsistency index (*I* ^2^). We will use the random effects model to calculate a pooled estimate.

**Discussion:**

Although several studies have shown that NAC can ameliorate hyperglycaemia-induced oxidative damage within the heart, this systematic review will be the first pre-registered synthesis of data to identify the cardioprotective potential of NAC against hyperglycaemia-induced oxidative damage. This result will help guide future research evaluating the cardioprotective role of NAC against DCM and better identify possible mechanisms of action for NAC to prevent oxidative damage with a diabetic heart.

**Systemic review registration:**

PROSPERO CRD42017055851.

**Electronic supplementary material:**

The online version of this article (doi:10.1186/s13643-017-0493-8) contains supplementary material, which is available to authorized users.

## Background

Diabetes mellitus (DM) is a chronic metabolic disease that is characterised by raised blood glucose levels. DM may arise due to insufficient production of insulin by the pancreatic beta-cells and is termed type 1 DM or the inability of the body to use the insulin it produces, which is known as type 2 DM [1]. The latter is related to obesity and represents approximately 90% of DM cases worldwide [1]. The increased percentage of premature deaths resulting from DM places a growing burden on healthcare resources in low- and middle-income countries such as South Africa [1]. For example, a combination of DM and cardiovascular diseases (CVDs) alone are estimated to contribute up to 24% of the 43% disease burden caused by non-communicable diseases in South Africa [2]. It is further known that almost 80% of deaths in diabetic patients are caused by cardiovascular-related complications [1, 3]. Although coronary artery disease (CAD) is the major cause of cardiovascular complications in diabetic patients, DM can also affect cardiac structure and function in the absence of CAD, a condition identified as diabetic cardiomyopathy (DCM) [4]. DCM is a unique clinical entity that is characterised by impaired myocardial substrate metabolism, left ventricular (LV) hypertrophy and diastolic dysfunction [4–6]. At present, there is no specific treatment for DCM; however, traditional treatment of heart failure and DM are the preferred methods used to contain DCM [7].

First-line antidiabetic drugs such as metformin and insulin can prolong the lives of diabetic patients by lowering glucose levels and have been shown to display cardioprotective properties [8–10]. However, long-term exposure to persistent hyperglycaemia appears to limit the cardioprotective effect of these drugs as shown by an increasing number of CVD-related deaths within diabetic patients [11]. Current consensus is that hyperglycaemia generates overproduction of free-radical species, which leads to oxidative stress and subsequent myocardial damage. Hyperglycaemic-induced oxidative stress is believed to directly cause modifications in cardiac structure and function that may occur in the late stage of DM [12]. Therefore, the proposed use of antioxidant therapies to curb intracellular oxidative damage and enhance the effect of current antidiabetic agents is among the leading hypothesis being tested to reduce the risk of myocardial infarction in diabetic patients [13–19].

*N*-acetyl cysteine (NAC) has emerged as a strong agent that is increasingly studied for its cardioprotective properties [13–19]. NAC is well absorbed by the intestine, and its supplementation presents strong antioxidant properties that are essential in preventing oxidative damage [20, 21]. NAC acts as a precursor to the synthesis of glutathione, which is one of the most important antioxidants. In experimental settings, NAC displays a strong potential to ameliorate oxidative stress resulting in reduced LV myocardial fibrosis and remodelling within a diabetic state [22, 23]. Although increasing studies are available on the cardioprotective properties of NAC [13–24], none of them have delivered a systematic or meta-analysis on its effect in preventing hyperglycaemia-induced oxidative damage that may result in the development of DCM.

### Objectives

The aim of this study is to systemically assess results of published data and summarise the state of knowledge on the cardioprotective potential of NAC against hyperglycaemia-induced oxidative damage that may lead to the development of DCM.

## Methods

### Study design

The preferred reporting system will follow PRISMA-P (Preferred Reporting Items for Systematic Review and Meta-Analysis Protocols) 2015 guidelines (Additional file 1). This protocol was registered with the International Prospective Register of Systemic Reviews (PROSPERO): CRD42017055851.

### Search strategy

The following electronic databases, with the help of an experienced librarian, will be searched from 1960, roughly corresponding to the time NAC was discovered to be an effective mucolytic agent [25], to March 2017: EMBASE, Cochrane Library, PubMed and Google scholar. The search will be based on key words and Medical Subject Headings (MeSH) terms such as ‘*N*-acetyl cysteine’, ‘diabetes mellitus’, ‘oxidative stress’ and ‘cardiovascular disease’, including corresponding synonym and associated terms for each item (Additional file 2). The search will be adopted to each database to eliminate any inconsistencies or de-duplication of references that may affect data extraction. References will be managed using EndNote.

### Study selection

In vitro and in vivo studies to be included are those that report on the use of NAC in various cardiovascular systems (either cardiac cells or animals) as monotherapy or in combination with another antioxidant or a known antidiabetic or cardioprotective agent to prevent or protect against (1) hyperglycaemia- or high glucose-induced oxidative damage; (2) oxidative stress; and (3) DCM. In addition, if available, studies reporting on the cardioprotective effect of NAC in diabetic patients (clinical trials or observational studies) of all ages will be included. In studies which have not yet reported the relevant outcome, data will be classified in a systematic review as ongoing studies. Each study that is included is expected to have a non-treated control group that received placebo. Types of publications from which data will be screened include original articles, editorials, letters, and articles from the grey literature (e.g. pre-prints and conference proceedings), whilst narrative reviews will only be screened for primary studies. The search will be restricted to studies written in English.

### Data extraction

Two investigators (P.V.D. and S.C.D.) will independently review all relevant articles and identify eligible studies. Disagreements or uncertainties will be resolved by consensus, whilst in case of persistent disagreement, a third reviewer will be consulted (B.B.N.). To extract relevant data for this review, a structured form that contains the following information will be created: first author’s details (name and year of publication), country of origin, type of study or model used, dose of NAC and of any other combinational agent used, duration of treatment, sample size (e.g. number of animals used per treatment group), assays performed and statistical analysis method used for each study. In addition, if any clinical trials are involved, relevant information such as participants’ characteristics, including age, gender, and diabetes profile (baseline blood glucose levels) will be extracted and recorded separately from the in vitro and in vivo data. Different type of studies, including in vitro, in vivo animals, healthy humans and diabetic humans will be analysed separately. Information extracted will be assessed to remove any duplicates that may exist. Moreover, the Covidence online tool [26] will be used to facilitate the process of screening, data extraction and analysis. The primary outcome of the study will be to determine whether NAC administration can protect the heart against hyperglycaemia-induced oxidative damage, whereas the secondary outcome will be to assess whether NAC administration can improve the efficacy of current antidiabetic or cardioprotective agents in protecting the heart against hyperglycaemia-induced oxidative damage.

### Quality assessment

The Cochrane Risk of Bias Assessment Tool will only be used to assess risk of bias in included Randomised Controlled Trials [27]. This tool contains several domains, including selection bias, performance bias, detection bias, attrition bias, reporting bias and other bias that may not be covered by the other domains. As explained by Foster et al., each endpoint and the risk of bias will be assessed individually to generate an overall score [28]. The Joanna Briggs Institute (JBI) Critical Appraisal tools for use in JBI Systematic Reviews, which is a specific checklist for non-randomised experimental studies, will be used for quality assessment of other types of studies [29]. This appraisal tool will assess the methodological quality of each study to determine the extent to which it addressed the possibility of bias in its design, conduct and analysis. Furthermore, the strength of evidence will be assessed and reported using the GRADE system [30]. GRADE addresses several apparent shortcomings of existing models of evidence evaluation. This includes assessment of methodological flaws within the component studies, consistency of results across diverse studies, precision of effect estimates, risk of publication bias and how effective the treatments have been shown to be.

### Data synthesis and analysis

To establish the cardioprotective effects of NAC on hyperglycaemic-induced oxidative damage, the adjusted risk estimates and 95% confidence intervals (CIs) will be calculated for each study. Adjusted risk estimates across studies will be pooled using random effects models with inverse variance weighting as recommended in the Cochrane handbook [31]. For synthesis, when studies cannot be combined for meta-analysis due to diversity of interventions, narrative synthesis will be conducted following guidelines by Popay et al. [32]. Inconsistency in the cardioprotective effects of NAC across different studies (heterogeneity) will be assessed using Cochrane Q statistic and the inconsistency index (*I* ^2^) [33], with an *I* ^2^ statistic of 0 and 50% indicating no heterogeneity and moderate heterogeneity, respectively. In addition to overall effect assessment, subgroup and sensitivity analyses will be conducted for types of participants (healthy patients or such with DM) for included human studies. Furthermore, studies evaluating NAC monotherapy groups will be analysed separately from studies specifically evaluating NAC combination therapy groups. All analyses will be conducted using Cochrane Review Manager 5.3 [34].

## Discussion

This systemic review will assess the cardioprotective intervention of NAC against diabetes-associated complications. We will specifically focus on reviewing literature on the protective potential of NAC against hyperglycaemia-induced oxidative damage related to the development of DCM. The proposal is made that persistent hyperglycaemia and resultant oxidative stress directly cause myocardial LV wall thickness and structural remodelling leading to diastolic dysfunction within a diabetic state [4–6, 12]. These are all conspicuous signs of DCM and are experimentally targeted by various pharmaceutical interventions in order to prolong the lives of diabetic individuals [35, 36].

Accumulative data is increasingly reporting on the cardioprotective properties of various pharmacological products in the treatment and prevention of DM and its complications [35–38]. Recently, our group has explored phytochemical compounds for their protective effect against DCM in cultured cardiomyocytes and diabetic mice [37–40]. Data from these studies has demonstrated that the antioxidant properties from these bioactive compounds when used either alone or in combination with metformin are important in combating hyperglycaemia-induced oxidative damage. Therefore, this has led us to hypothesise that nutraceuticals and pharmacological compounds with strong antioxidant properties such as NAC may be the key in preventing hyperglycaemia-induced oxidative damage within the heart and improving the lives of diabetic individuals. To date, several studies have already assessed the protective activity of NAC against DCM-associated complications [13–24]; however, none have delivered an extensive overview about the topic.

In summary, this systemic review will address the existing knowledge gap regarding the protective effect of NAC against DCM, in particular, for the prevention of hyperglycaemia-induced oxidative damage. An extensive synthesis of the available data will permit identification of evidence gaps. Furthermore, since NAC is already listed in the World Health Organisation’s list of essential medicines, this review may determine whether more research is necessary to establish its use as a protective therapy against DCM.

## Additional files

Additional file 1: PRISMA-P (Preferred Reporting Items for Systemic review and Meta-Analysis Protocols). Recommended items to address in a systemic review protocol.

Additional file 2: Search strategy.

## Abbreviations

CAD: Coronary artery disease
CVD: Cardiovascular disease
DCM: Diabetic cardiomyopathy
DM: Diabetes mellitus
JBI: Joanna Briggs Institute
LV: Left ventricular
NAC: *N*-acetyl cysteine
PRISMA-P: Preferred Reporting Items for Systematic Review and Meta-Analysis Protocols
