# Supplementary material for: Cardioprotective potential of N-acetyl cysteine against hyperglycaemia-induced oxidative damage: a protocol for a systematic review
Source: Syst Rev. 2017 May 12;6:96. doi: 10.1186/s13643-017-0493-8 (PMC5427588; doi:10.1186/s13643-017-0493-8)
Supplement: Supplementary file 2 — Search strategy. [file 13643_2017_493_MOESM2_ESM.docx]

**Additional file 2: Search strategy run 03 March 2017**

| **Concept 1:**  **N acetyl cysteine** | **Synonyms to be searched (MeSH OR textwords)** | |
| --- | --- | --- |
| **PubMed** (hits= 17805)  ("acetylcysteine"[MeSH Terms] OR "acetylcysteine"[All Fields] OR "n acetyl cysteine"[All Fields]) | Acemuc | Acebraus |
|  | Acetabs | Acétylcystéine GNR |
|  | Acetylcystein AL | Acetylcysteine, monoammonium salt |
|  | Acetylcystein Atid | Acetylin |
|  | Acetylcystein Heumann | Acetyst |
|  | Acetylcystein Trom | Airbron |
|  | Acetylcystein, mentopin | Alveolex |
|  | Acetylcysteine Hydrochloride | Azubronchin |
|  | Acetylcysteine Sodium | Bisolvon NAC |
|  | Acetylcysteine Zinc | Bromuc |
|  | Acetylcysteine, (D)-Isomer | Broncho Fips |
|  | Acetylcysteine, (DL)-Isomer | BronchoFips |
|  | Acetylcysteine, Monosodium Salt | Broncho-Fips |
|  | Acid, Mercapturic | Broncholysin |
|  | Fabrol | Broncoclar |
|  | Fluimucil | Codotussyl |
|  | Fluprowit | Cystamucil |
|  | Genac | Dampo Mucopect |
|  | Hydrochloride, Acetylcysteine | Durabronchal |
|  | Jenacystein | Eurespiran |
|  | Jenapharm | Exomuc |
|  | Lantamed | Frekatuss |
|  | Lindocetyl | Hoestil |
|  | M Pectil | Hustengetränk, Optipect |
|  | mentopin Acetylcystein | Ilube |
|  | Mercapturic Acid | Larylin NAC |
|  | Monosodium Salt Acetylcysteine | Monoammonium salt acetylcysteine |
|  | MPectil | Muco Sanigen |
|  | M-Pectil | Mucomyst |
|  | Muciteran | Mucopect, Dampo |
|  | N Acetyl L cysteine | Mucosil |
|  | N Acetylcysteine | Mucosol |
|  | NAC AL | Mucosolvin |
|  | NAC Zambon | NAC, Bisolvon |
|  | N-Acetylcysteine | N-Acetyl-L-cysteine |
|  | Sodium, Acetylcysteine | Optipect Hustengetränk |
|  | Solmucol | Sanigen, Muco |
|  | Zambon, NAC | Siccoral |
|  | Zinc, Acetylcysteine | Siran |

| **Concept 2:**  **Diabetes mellitus** | **Associated words to be searched (MeSH OR textwords)** |
| --- | --- |
| **PubMed** (hits= 413348)  ("diabetes mellitus"[MeSH Terms] OR ("diabetes"[All Fields] AND "mellitus"[All Fields]) OR "diabetes mellitus"[All Fields]) | Diabetes mellitus |
|  | Glucose metabolism disorders |
|  | Hyperglycaemia |
|  | Hyperglycemia |
|  | Metabolic diseases |
|  |  |

| **Concept 3:**  **Oxidative stress** | **Associated words to be searched (MeSH OR textwords)** |
| --- | --- |
| **PubMed** (hits= 169638)  ("oxidative stress"[MeSH Terms] OR ("oxidative"[All Fields] AND "stress"[All Fields]) OR "oxidative stress"[All Fields]) | Free radical species |
|  | Oxidative stress |
|  | Oxidative stresses |
|  | Reactive nitrogen species |
|  | Reactive oxygen species |
|  | Stress, oxidative |
|  | Stresses, oxidative |

| **Concept 4:**  **Cardiovascular disease** | **Associated words to be searched (MeSH OR textwords)** |
| --- | --- |
| **PubMed** (hits= 2202372)  ("cardiovascular diseases"[MeSH Terms] OR ("cardiovascular"[All Fields] AND "diseases"[All Fields]) OR "cardiovascular diseases"[All Fields] OR ("cardiovascular"[All Fields] AND "disease"[All Fields]) OR "cardiovascular disease"[All Fields]) | Cardiomyocytes |
|  | Cardiomyopathy |
|  | Cardiovascular disease |
|  | Diabetic cardiomyopathy |
|  | Heart cells |
|  | Heart disease |
|  | Heart failure |
|  | Myocardial infarction |
|  |  |
|  |  |
|  |  |
|  |  |

Combine Concept 1 AND Concept 2 AND Concept 3 AND Concept 4
